# Lentinan-Based Multifunctional Composite Hydrogel for Wound Healing

**DOI:** 10.3390/gels12080716

**Published:** 2026-08-13

**Authors:** Niu Liu, Wengui Xu, Xinmiao He, Jinwang Zou, Jinhao Pan, Chenyi Feng, Yao Wu, Changchun Li, Hairong Xiong, Zongbao Zhou

**Affiliations:** Hubei Key Laboratory of Resource Utilization and Quality Control of Characteristic Crops, College of Life Science and Technology, Hubei Engineering University, Xiaogan 432000, China; liuniu1221@126.com (N.L.); m15870787121@163.com (W.X.); 13296693616@163.com (X.H.); 19371567729@163.com (J.Z.); 18585448336@163.com (J.P.); 18040509631@163.com (C.F.); wuyao927@163.com (Y.W.); lcc386@163.com (C.L.)

**Keywords:** tissue-adhesive hydrogel, lentinan, tannic acid, wound dressing

## Abstract

Conventional wound dressings suffer from unbalanced tissue adhesion, skin-mismatched mechanical properties, and insufficient long-term bioactivity. Herein, a dual physically cross-linked multifunctional tissue adhesive lentinan–polyvinyl alcohol (PVA)–tannic acid membrane (LPTM) was fabricated via a freeze–thaw-assisted immersion strategy. The optimized LPTM-3 hydrogel patch, with a stable network formed by PVA crystalline cross-linking and hydrogen bond cross-linking, achieved a balanced tensile strength of 16.1 MPa and an elongation at break of 149.3%, robust yet non-damaging porcine skin adhesion of 163.17 kPa, antibacterial activity, and good biocompatibility with a hemolysis rate below 2.24%. Notably, LPTM-3 promoted fibroblast migration and modulated inflammatory cytokine expression in vitro, and achieved 99.06% full-thickness wound closure in rats on day 14, matching a commercial dressing. This work provides a promising candidate for clinical wound repair and a facile design strategy for polysaccharide-based bioadhesive materials.

## 1. Introduction

The skin, the largest organ of the human body, acts as a critical physical barrier against exogenous pathogen invasion, body fluid homeostasis maintenance, and physiological metabolism regulation [1]. Full-thickness skin defects caused by acute trauma, burns, surgery, and chronic ulcers (e.g., diabetic foot, pressure sores) remain a major global clinical challenge, inducing severe pain, high infection risk, and even life-threatening systemic sepsis in severe cases [2]. Wound dressings are the core intervention for skin defect repair, and an ideal dressing requires multiple synergistic properties to support the complex healing process. Among these, balanced tissue adhesiveness is the primary prerequisite for clinical use: robust yet atraumatic adhesion ensures stable fixation on dynamic wound surfaces, avoids dressing displacement and secondary bacterial invasion, and prevents mechanical injury during replacement [3]. Meanwhile, the dressing should integrate skin-matched mechanical properties, long-lasting antibacterial and antioxidant activity, excellent biocompatibility, and controllable biodegradation to maintain a favorable wound microenvironment [4]. However, widely used clinical dressings cannot simultaneously meet these requirements, greatly limiting their long-term application in complex wound repair.

In recent years, three-dimensional hydrophilic hydrogel dressings have attracted extensive attention in wound repair for their extracellular matrix-mimetic structure, excellent water retention, tunable properties, and good biocompatibility [5]. Compared with synthetic polymer hydrogels, natural polysaccharide-based ones are widely studied for their natural origin, low immunogenicity, inherent bioactivity, and biodegradability. Among them, lentinan (LNT), a β-1,3-glucan from Lentinus edodes, exhibits excellent biocompatibility, immunomodulatory, antioxidant, and pro-proliferative activities, with broad biomedical prospects [6,7]. However, pure LNT has poor gel-forming ability and can barely form stable hydrogels, while existing modifications mostly rely on toxic chemical crosslinkers that induce cytotoxicity and inflammation in vivo. Tannic acid (TA) is a natural polyphenol with abundant phenolic hydroxyl groups. Our previous study confirmed that TA-functionalized LNT hydrogels achieved enhanced tissue adhesion, robust antibacterial activity, and accelerated infected wound healing, preliminarily validating the wound care potential of LNT-based systems [8]. Nevertheless, single-component LNT hydrogels still have weak mechanical properties, insufficient adhesion, and limited function, failing to meet the demands of dynamic wound environments and restricting their clinical translation.

To overcome the gel-forming defects of LNT and construct adhesive multifunctional hydrogel systems, polyvinyl alcohol (PVA) was selected for synergistic modification. PVA is an FDA-approved medical synthetic polymer with excellent biocompatibility and film-forming ability [9]. Guo et al. developed a highly stretchable and adhesive PVA-based hydrogel dressing for joint wound healing, confirming that PVA-based systems can be structurally tuned to match the mechanical and adhesive requirements of dynamic wound environments [10]. Through repeated freeze–thaw cycles, PVA molecular chains can form a physically cross-linked network via crystalline regions, enabling the fabrication of hydrogels with stable mechanical properties without chemical crosslinkers and avoiding associated safety risks [11]. Nevertheless, pure PVA hydrogel lacks inherent tissue adhesiveness, antibacterial activity, and bioactivity, making it unsuitable for standalone use as a functional wound dressing. Dong’s team constructed a PVA-sodium alginate-TA composite hydrogel, demonstrating that TA can act as a physical crosslinker via multi-site hydrogen bond interactions to enhance the mechanical strength and tissue adhesion of PVA-based hydrogels [12]. Subsequent studies further confirmed that TA-crosslinked PVA hydrogels could be endowed with excellent self-healing and antibacterial properties [13], and TA-based PVA dual-network hydrogels could achieve efficient antibacterial activity and accelerated infected wound healing [14], fully verifying the application potential of TA-PVA systems in wound dressings. Notably, existing LNT-based hydrogels predominantly rely on chemical crosslinkers that pose cytotoxicity and inflammation risks, while reported PVA-TA systems lack the polysaccharide-derived bioactivity essential for active wound healing. The present study addresses this scientific gap by constructing the first fully physically cross-linked LNT-PVA-TA ternary system via a dual-network design strategy, achieving tissue adhesion, mechanical adaptability, sustained bioactive release, and accelerated wound healing without chemical crosslinker-associated safety concerns. We hypothesized that this ternary system could effectively overcome the core limitations of poor gel-forming ability of pure LNT and the non-adhesive, non-functional nature of pure PVA hydrogel; by tuning the LNT/PVA mass ratio, the adhesion, mechanical properties, degradation behavior, and bioactivity of the hydrogel can be precisely regulated to fabricate a multifunctional wound dressing.

Based on the above design strategy and scientific hypothesis, we herein carried out a systematic study to verify the feasibility of constructing a multifunctional adhesive wound dressing based on the proposed dual physically cross-linked system. First, a series of lentinan–polyvinyl alcohol–tannic acid membrane (LPTM) hydrogel dressings with gradient LNT/PVA mass ratios were fabricated via a freeze–thaw-assisted immersion method. The overall performance of different formulations was evaluated to screen the optimal candidate for follow-up investigations. We then systematically characterized the in vitro biological function and biosafety of the optimized hydrogel, and further assessed its in vivo wound healing efficacy via a full-thickness skin defect animal model to verify our scientific hypothesis. This study is expected to provide a promising multifunctional adhesive hydrogel candidate for clinical wound repair, and offer a facile design strategy for natural polysaccharide-based biomedical adhesive materials.

## 2. Results and Discussion

### 2.1. Fabrication and Characterization of LNT-PVA-TA Composite Hydrogel Patches

#### 2.1.1. Macroscopic Morphology

**Figure 1:**
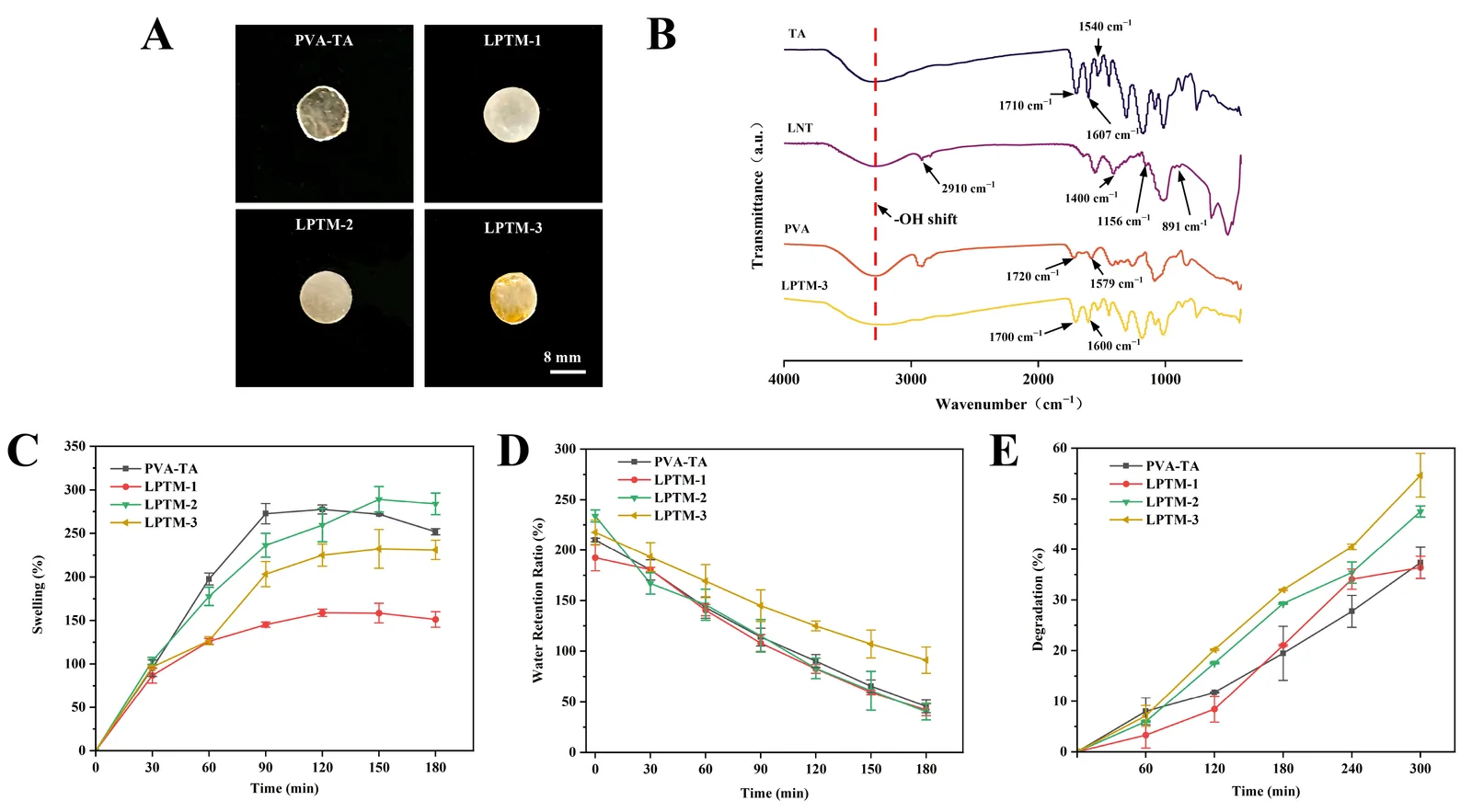
Macroscopic morphology of PVA−TA, LPTM−1, LPTM−2, and LPTM−3 hydrogels (**A**), FTIR spectra of TA, LNT, PVA and LPTM−3 (**B**), swelling behavior (**C**), water retention capacity (**D**), and degradation profile (**E**).

The macroscopic morphology of the hydrogel patches (Figure 1A) revealed a profound influence of Lentinan (LNT) content on structural uniformity and physicochemical properties. The control PVA-TA hydrogel exhibited an irregular, rough-edged shape with a dark brownish hue, indicative of inhomogeneous cross-linking. In contrast, all LPTM series hydrogels formed smooth, circular discs, demonstrating that LNT incorporation effectively improved moldability and structural homogeneity. Specifically, LPTM-1 (LNT:PVA = 1:3, *w*/*w*) displayed a pale white, uniform texture, suggesting a relatively balanced cross-linking network. As the LNT ratio increased to 1:1 (LPTM-2), the hydrogel maintained a light beige color with consistent texture. Further increasing the LNT proportion to 3:1 (LPTM-3) resulted in a slightly yellowish appearance, attributed to increased polysaccharide content and potential auto-oxidation of LNT during fabrication [15].

#### 2.1.2. FTIR Spectroscopy

FTIR spectroscopy was employed to characterize the chemical structure and intermolecular interactions in the LPTM-3 composite hydrogel, with the spectra of TA, LNT, and PVA analyzed as references (Figure 1B). For TA, a broad absorption band at 3200–3600 cm^−1^ was attributed to the stretching vibration of phenolic hydroxyl (-OH) groups. Distinct peaks at 1710 cm^−1^, 1607 cm^−1^, and 1540 cm^−1^ corresponded to the C=O stretching of carboxylic ester groups, the aromatic ring C=C stretching, and the benzene ring C=C skeletal vibration, respectively, consistent with the characteristic features of TA [16]. In the LNT spectrum, a peak at 2910 cm^−1^ was assigned to the C-H stretching vibration of sugar units. Characteristic polysaccharide peaks at 1400 cm^−1^ (C-H bending of sugar rings), 1156 cm^−1^ (C-O-C stretching of glycosidic linkages), and 891 cm^−1^ (a marker for β-glycosidic bonds in LNT) confirmed the structural integrity of the polysaccharide [17]. For PVA, peaks at 1720 cm^−1^ and 1579 cm^−1^ were observed, which may arise from the formation of hydrogen-bonded complexes during processing, alongside the broad -OH stretching band at ~3300 cm^−1^ typical of PVA [18]. In the LPTM-3 spectrum, significant changes confirmed successful composite formation: the broad -OH stretching peak shifted to a lower wavenumber compared to pure PVA, indicating strong hydrogen bonding between the hydroxyl groups of PVA/LNT and the phenolic hydroxyl/carboxyl groups of TA; new peaks at 1700 cm^−1^ and 1600 cm^−1^ appeared, corresponding to the C=O stretching and aromatic C=C stretching of TA, respectively, verifying the integration of TA into the composite network [19]. Meanwhile, the characteristic peaks of LNT and PVA were retained, confirming the successful incorporation of all three components without significant structural degradation. These observations collectively confirm that LPTM-3 was successfully synthesized via hydrogen bonding and intermolecular interactions, forming a stable multi-component cross-linked network.

#### 2.1.3. Swelling Behavior

Swelling behavior is a critical indicator of the hydrogel’s ability to absorb and retain aqueous media, which directly impacts biomedical applications such as wound dressing [20]. The swelling ratio of all hydrogel formulations increased rapidly within the first 90 min, followed by gradual stabilization (Figure 1C). The control PVA-TA hydrogel reached a maximum swelling ratio of 277.39 ± 5.10% at 120 min, which slightly decreased to 251.81 ± 3.57% by 180 min, indicating a relatively stable cross-linked network. In contrast, LPTM-1 exhibited the lowest swelling ratio, peaking at 158.95 ± 4.20% at 120 min and remaining stable thereafter. This reduced swelling can be attributed to enhanced cross-linking density at moderate LNT content, which restricts polymer network expansion. Notably, while LPTM-2 exhibited a slightly higher maximum swelling ratio than PVA-TA (289.12 ± 14.81% at 150 min), its rapid water loss (only 40.34% retained at 180 min) limited its utility for sustained moisture delivery. In sharp contrast, LPTM-3 maintained a high and stable swelling capacity (~230%) after 150 min, which is advantageous for sustained moisture absorption in long-term biomedical applications. The higher swelling capacity of LPTM-2 and LPTM-3 compared to LPTM-1 is attributed to the abundant hydroxyl groups in LNT, which enhance the hydrophilicity of the composite network and promote water absorption [21].

#### 2.1.4. Water Retention Capacity

Water retention capacity reflects the hydrogel’s ability to retain absorbed water under environmental stress, which is essential for maintaining a moist environment conducive to wound healing [22]. The initial water retention capacity followed the order: LPTM-2 (233.82 ± 5.97%) > LPTM-3 (217.66 ± 12.03%) > PVA-TA (210.06 ± 1.76%) > LPTM-1 (192.30 ± 12.92%), indicating that LNT incorporation initially enhanced water retention (Figure 1D). Over time, all hydrogels exhibited gradual water loss, but significant differences were observed in long-term retention. LPTM-3 retained a significantly higher water content (91.17 ± 12.94%) at 180 min compared to LPTM-2 (40.34 ± 8.26%), PVA-TA (45.61 ± 6.42%), and LPTM-1 (42.10 ± 5.75%), demonstrating superior long-term moisture retention. This enhanced retention in LPTM-3 stems from the balanced network structure formed at a 3:1 LNT:PVA ratio, which optimizes porosity and hydrophilicity to slow water evaporation [23]. While LPTM-1’s compact network slowed water evaporation to a certain extent, its absolute water content remained significantly lower than that of LPTM-3 at all time points, further highlighting LPTM-3’s superior long-term moisture retention capability.

#### 2.1.5. Degradation Behavior

The degradation profile is a key factor in determining the biocompatibility and suitability of hydrogels for temporary biomedical applications [24]. All hydrogels exhibited time-dependent degradation behavior, with degradation rates increasing with higher LNT content (Figure 1E). The control PVA-TA hydrogel showed the slowest degradation rate, reaching only 37.34 ± 3.10% at 300 min, which is attributed to its stable, densely cross-linked network. In contrast, LPTM-3 exhibited the highest degradation rate, reaching 54.66 ± 4.31% at 300 min, followed by LPTM-2 (47.48 ± 1.08%) and LPTM-1 (36.37 ± 2.20%). The accelerated degradation of the LPTM series hydrogels, particularly LPTM-3, is attributed to the enzymatic susceptibility of the polysaccharide backbone of LNT, which provides additional cleavage sites for hydrolytic enzymes. As the LNT ratio increased from 1:3 (LPTM-1) to 3:1 (LPTM-3), the degradation rate increased, indicating that higher LNT content enhances the biodegradability of the composite hydrogel—an essential property for reducing the risk of foreign body reactions in vivo. The in vitro degradation was conducted under accelerated enzymatic conditions, and the actual in vivo degradation rate in the wound environment is expected to be substantially slower due to lower and more variable enzyme concentrations.

#### 2.1.6. Mechanical Tensile Behavior

**Figure 2:**
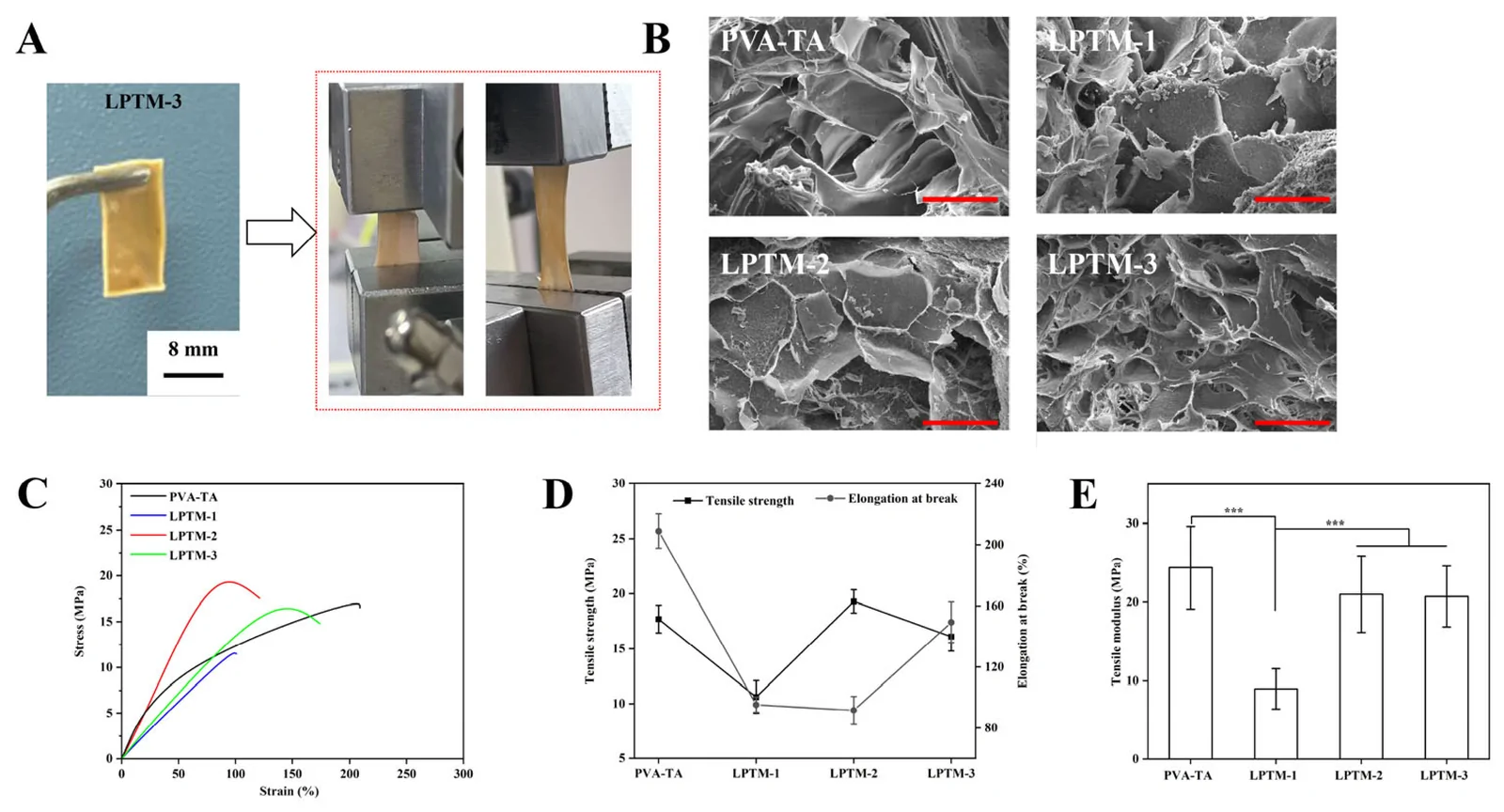
Representative photographs of LPTM-3 under tensile deformation (**A**), SEM images of the cross-sectional morphology of PVA−TA, LPTM−1, LPTM−2, and LPTM−3 hydrogels(scale bar = 200 μm) (**B**), stress–strain curves (**C**), tensile strength and elongation at break (**D**), and tensile modulus (**E**) of PVA−TA, LPTM−1, LPTM−2, and LPTM−3 hydrogels. Data are presented as mean ± standard deviation (*n* = 3); *** *p* < 0.001.

The mechanical performance of the LPTM composite hydrogels was systematically evaluated, as flexible biomedical devices require sufficient strength and ductility to withstand dynamic deformation. Representative photographs (Figure 2A) demonstrate the excellent flexibility and processability of LPTM-3, which could be fabricated into a free-standing patch and withstand significant tensile deformation without catastrophic fracture—a critical attribute for wearable biomedical devices [25].

The cross-sectional morphology of the hydrogels was further characterized by SEM (Figure 2B). SEM samples were prepared by freeze-drying to preserve the hydrated pore architecture. Before freeze–thaw cycling, the cast films exhibited a dense, non-porous cross-sectional morphology, confirming that the porous structure was induced by ice crystal-templated PVA crystallization during freeze–thaw treatment [26]. The PVA-TA hydrogel exhibited a dense, compact structure with minimal porosity, whereas the LPTM series hydrogels displayed a distinct porous microstructure with interconnected pores formed during the freeze–thaw process. As the LNT content increased from LPTM-1 to LPTM-3, the pore size gradually increased and the network became more open, suggesting that LNT incorporation modulated the crystallization behavior of PVA during freeze–thaw cycling and promoted the formation of a more porous architecture. This porous structure is beneficial for nutrient diffusion and cellular infiltration in wound healing applications. The rheological characterization was not performed in this study and will be included in future investigations to provide additional insight into the viscoelastic behavior of the hydrogel network.

The stress–strain curves (Figure 2C) provided a qualitative overview of the mechanical responses, while quantitative analysis of tensile strength and elongation at break (Figure 2D) revealed the critical role of LNT content in modulating mechanical properties. The control PVA-TA hydrogel exhibited a tensile strength of 17.7 ± 1.26 MPa and an elongation at break of 208.9 ± 11.2%, reflecting a densely cross-linked network with high tensile strength, while its excessively high tensile modulus (24.38 ± 5.26 MPa) leads to poor flexibility, limiting its suitability for dynamic, flexible applications. In contrast, LPTM-1 (LNT:PVA = 1:3) showed significant reductions in both tensile strength (10.6 ± 1.5 MPa, ~40% lower than PVA-TA) and elongation at break (95.3 ± 5.1%, ~54% lower than PVA-TA), indicating that moderate LNT incorporation disrupted the compact PVA-TA network, weakening intermolecular interactions and leading to a more brittle structure [27]. Increasing the LNT ratio to 1:1 (LPTM-2) restored and even enhanced tensile strength to 19.3 ± 1.05 MPa (~9% higher than PVA-TA), likely due to the formation of additional hydrogen bonds between LNT, PVA, and TA that reinforced the network; however, this came at the cost of ductility, with elongation at break dropping to 91.7 ± 9.1% (~4% lower than LPTM-1). This reduction in ductility makes LPTM-2 less suitable for dynamic applications where repeated deformation is required, unlike LPTM-3, which maintains both rigidity and flexibility to withstand wearable or implantable stresses. Notably, LPTM-3 (LNT:PVA = 3:1) achieved a favorable balance: while its tensile strength (16.1 ± 1.3 MPa) was slightly lower than that of PVA-TA (~9% reduction) and LPTM-2 (~16% reduction), its elongation at break (149.3 ± 13.3%) was significantly higher than both LPTM-1 (~57% increase) and LPTM-2 (~63% increase), indicating improved ductility and resilience from a more balanced network structure, where LNT modulates the network to enhance flexibility without severely compromising strength.

Analysis of tensile modulus (Figure 2E) further clarified structure–property relationships, with quantitative data revealing distinct rigidity profiles across formulations: PVA-TA exhibited the highest tensile modulus (24.38 ± 5.26 MPa), reflecting its rigid, densely cross-linked network, which resists deformation effectively but may be overly rigid for applications requiring repeated mechanical stress. LPTM-1 showed the lowest modulus (8.97 ± 2.57 MPa), consistent with the disrupted network structure observed in Figure 2D, where LNT incorporation at a 1:3 ratio disrupted the original PVA-TA network, weakening intermolecular forces and reducing resistance to deformation. LPTM-2 exhibited a modulus of 21.01 ± 4.86 MPa, indicating high rigidity and a tightly cross-linked network, which aligns with its high tensile strength but low ductility. In contrast, LPTM-3 exhibited a modulus of 20.73 ± 3.86 MPa, which is comparable to LPTM-2 and only slightly lower than PVA-TA, yet it maintained a significantly higher elongation at break (149.3% vs. 91.7% for LPTM-2). This unique combination of comparable modulus and superior ductility indicates that LPTM-3’s network structure is optimized: the 3:1 LNT:PVA ratio promotes sufficient intermolecular interactions to maintain rigidity via hydrogen bonding while allowing molecular chains to undergo significant deformation, striking a critical balance between strength, flexibility, and resilience [28]. These results highlight that LNT content can be tailored to modulate mechanical performance: low LNT ratios disrupt the network and reduce performance, while higher ratios enhance strength but sacrifice ductility. Notably, LPTM-3 emerges as the most promising candidate for flexible biomedical applications requiring both structural integrity and deformability, as it achieves an optimal balance between mechanical rigidity and toughness.

#### 2.1.7. Adhesive Strength on Porcine Skin

**Figure 3:**
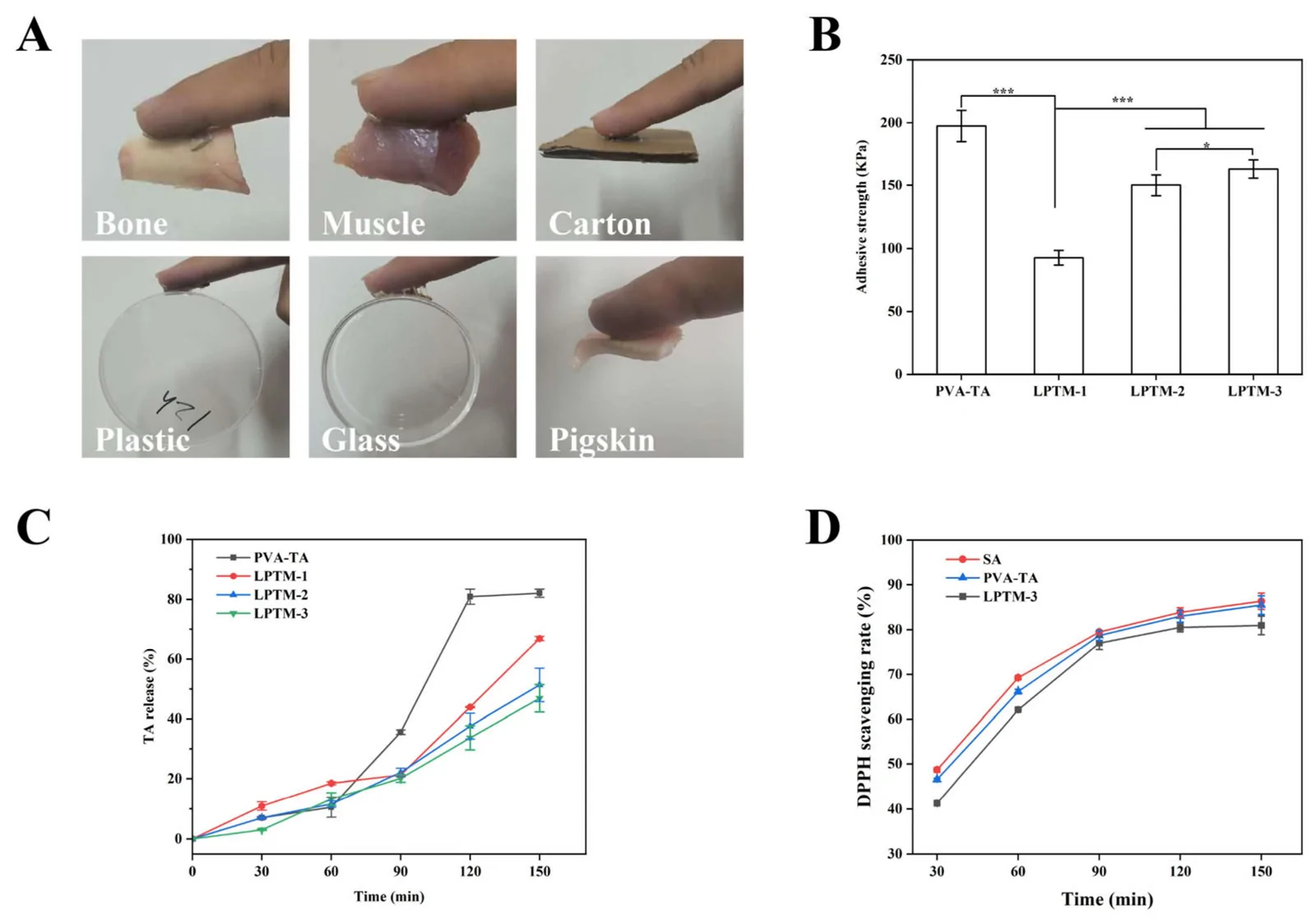
Representative photographs of LPTM−3 adhering to diverse surfaces (**A**), adhesive strength on porcine skin (**B**), TA release kinetics (**C**), and DPPH radical scavenging activity of SA, PVA−TA, and LPTM−3 (**D**). Data are presented as mean ± standard deviation (*n* = 3); * *p* < 0.05, *** *p* < 0.001.

For biomedical patches, robust adhesion to biological tissue, while avoiding excessive adhesion that causes secondary damage, is essential [29]. The adhesive performance of the LPTM composite hydrogels was first evaluated on porcine skin and further demonstrated on diverse materials (Figure 3A,B). Representative photographs (Figure 3A) confirm that LPTM-3 can firmly adhere to multiple surfaces, including bone, muscle, carton, plastic, glass, and porcine skin, highlighting its versatile applicability for biomedical and flexible device use. Quantitative analysis of adhesive strength on porcine skin (Figure 3B) revealed distinct differences across formulations: the control PVA-TA hydrogel exhibited the highest adhesive strength (197.50 ± 12.56 kPa), attributed to dense hydrogen bonding between PVA-TA hydroxyl groups and the collagen-rich porcine skin surface. However, this excessive adhesion may cause pain or tissue damage when the patch is removed [30]. In contrast, LPTM-1 (LNT:PVA = 1:3) showed a significant reduction in adhesive strength to 92.72 ± 5.70 kPa (~47% of PVA-TA), indicating that moderate LNT incorporation disrupted the original PVA-TA network, weakening interfacial hydrogen bonding and reducing surface contact—too weak for stable tissue adhesion. Increasing the LNT ratio to 1:1 (LPTM-2) and 3:1 (LPTM-3) restored adhesive strength to 150.23 ± 8.23 kPa (~76% of PVA-TA) and 163.17 ± 7.23 kPa (~83% of PVA-TA), respectively. This recovery stems from additional hydroxyl groups in LNT, which re-establish hydrogen bonds with both the porcine skin substrate and the PVA-TA matrix. Notably, LPTM-3 exhibited slightly higher adhesive strength than LPTM-2, suggesting that the 3:1 LNT:PVA ratio optimizes the balance between network cohesion and interfacial adhesion—avoiding brittleness associated with excessive cross-linking while maintaining robust bonding to biological tissue, and avoiding the excessive adhesion of PVA-TA that risks secondary damage.

#### 2.1.8. TA Release Kinetics

The release of TA is critical for the bioactivity of the hydrogel patches (e.g., antioxidant, anti-inflammatory, and antibacterial effects), and its kinetics were monitored over 150 min (Figure 3C). All formulations exhibited time-dependent TA release, with distinct profiles modulated by LNT content. The control PVA-TA hydrogel showed rapid initial release, reaching 80.90 ± 2.53% at 120 min, followed by saturation at 82.07 ± 1.36% by 150 min. This burst release is attributed to TA molecules being loosely bound at the hydrogel surface, while the dense PVA-TA network restricts deeper TA diffusion in later stages—this burst may cause local cytotoxicity due to high initial TA concentrations, limiting its biomedical applicability [31]. LPTM-1 (LNT: PVA = 1:3) displayed slower release, with only 66.90 ± 0.71% released by 150 min, likely due to increased cross-linking density that hinders molecular mobility and TA diffusion—too slow to achieve effective therapeutic concentrations in a timely manner. As LNT content increased to 1:1 (LPTM-2) and 3:1 (LPTM-3), release rates became more gradual: LPTM-2 reached 51.31 ± 5.61% and LPTM-3 reached 46.86 ± 4.59% by 150 min, with no abrupt burst release. The TA release profiles were further analyzed using four kinetic models (Table 1) [32]. The zero-order model provided the best fit for all formulations (r^2^ = 0.90–0.97), suggesting a near-constant release rate. The Higuchi model also showed reasonable fits (r^2^ = 0.83–0.92), indicating a diffusion contribution. In contrast, the first-order model showed the lowest fit quality (r^2^ = 0.82–0.93), ruling out concentration-driven release. The Korsmeyer–Peppas exponent n ranged from 1.07 to 1.72, pointing to Super Case II transport dominated by polymer swelling and network relaxation, which is consistent with the dynamic hydrogen-bonded PVA–TA–LNT network. Notably, the release rate constant k_0_ decreased progressively from PVA-TA (0.6257) to LPTM-3 (0.3058), and the cumulative release at 150 min dropped from 82.1% (PVA-TA) to 46.9% (LPTM-3), confirming that LNT incorporation effectively retards TA release through additional physical cross-linking.

Based on the comprehensive characterization of structural, physical, mechanical, and functional properties of all hydrogel formulations, LPTM-3 was confirmed as the optimal formulation for subsequent bioactivity evaluations. This conclusion is supported by the following key findings: In terms of structural stability, LPTM-3 formed a stable multi-component cross-linked network via hydrogen bonding between PVA, LNT, and TA (verified by FTIR), avoiding the inhomogeneous cross-linking of the control PVA-TA hydrogel and the network disruption of LPTM-1. Its slightly yellowish, smooth, circular morphology also indicated excellent moldability, which is essential for fabricating uniform biomedical patches. For physical properties, LPTM-3 maintained a high and stable swelling capacity (~230%) for sustained moisture absorption, coupled with superior long-term water retention (91.17% at 180 min)—a critical combination for creating a moist microenvironment conducive to wound healing. In contrast, PVA-TA showed rapid water loss, LPTM-1 had insufficient swelling, and LPTM-2 exhibited rapid water loss despite high initial swelling, all limiting their long-term utility. In mechanical performance, LPTM-3 struck a critical balance between strength and ductility: its tensile strength (16.1 ± 1.3 MPa) and modulus (20.73 ± 3.86 MPa) were comparable to PVA-TA and LPTM-2, ensuring structural integrity, while its significantly higher elongation at break (149.3 ± 13.3%) enabled it to withstand dynamic deformation—essential for wearable or implantable biomedical devices. LPTM-1 was too brittle, and LPTM-2 lacked ductility, making them unsuitable for dynamic applications. For functional properties (adhesion and TA release), LPTM-3 exhibited optimal adhesive strength (163.17 ± 7.23 kPa) on porcine skin—robust enough for stable tissue bonding but avoiding the excessive adhesion of PVA-TA that risks secondary damage. Its sustained TA release kinetics eliminated burst release, ensuring prolonged bioavailability without local cytotoxicity, while LPTM-1’s release was too slow and PVA-TA’s release was too rapid. Additionally, LPTM-3’s degradation rate (54.66 ± 4.31% at 300 min) was optimal for temporary biomedical applications, ensuring gradual degradation as tissue heals, unlike the slow degradation of PVA-TA and LPTM-1. LPTM-3’s stable release kinetics suggest a well-tuned network structure that balances porosity and cross-linking, enabling a more gradual initial release profile that avoids burst-associated cytotoxicity.

### 2.2. Antioxidant Activity

Given that LPTM-3 was confirmed as the optimal formulation, its antioxidant performance was evaluated via DPPH radical scavenging, with salicylic acid (SA) as a reference (Figure 3D)—antioxidant activity is critical for mitigating oxidative stress in wound healing, as excessive reactive oxygen species (ROS) delay tissue regeneration and promote inflammation [33]. All three groups exhibited time-dependent scavenging activity. At 30 min, SA achieved the highest scavenging rate (48.67 ± 0.53%), followed by PVA-TA and LPTM-3 (41.26 ± 0.53%). By 150 min, SA reached 86.37 ± 1.82%, LPTM-3 reached 84.97 ± 2.09%, and PVA-TA reached a comparable level, indicating that the antioxidant efficacy of both hydrogel formulations was primarily driven by TA-mediated ROS scavenging. Notably, LPTM-3 exhibited slightly lower scavenging rates than PVA-TA at early time points, which is consistent with its more gradual TA release profile (Figure 3C); the sustained rather than burst release of TA from LPTM-3 is expected to provide prolonged antioxidant protection over extended periods, as opposed to the rapid initial scavenging followed by depletion observed in PVA-TA. These results demonstrate that LPTM-3 can provide sustained oxidative stress protection, a critical attribute for applications such as wound healing, where prolonged antioxidant activity supports tissue regeneration and reduces inflammation.

### 2.3. Antibacterial Activity

**Figure 4:**
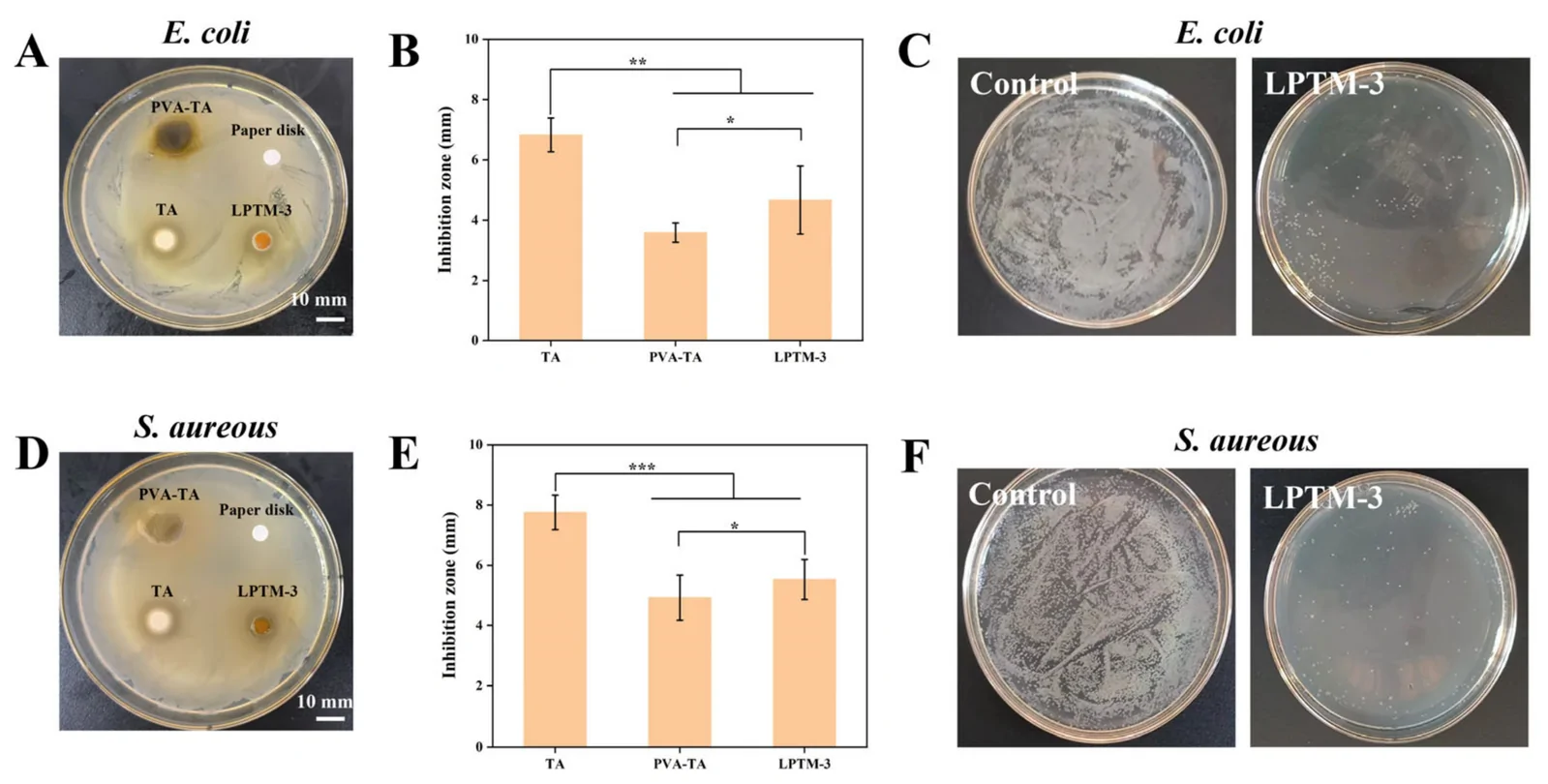
Antibacterial activity of LPTM-3 against *E. coli* and *S. aureus*. Representative images of inhibition zones formed by paper disk, PVA−TA, TA, and LPTM−3 against *E. coli* (**A**) and *S. aureus* (**D**) (scale bar = 10 mm). Quantitative analysis of inhibition zone diameters for *E. coli* (**B**) and *S. aureus* (**E**). Direct contact antibacterial efficacy of LPTM−3 against *E. coli* (**C**) and *S. aureus* (**F**), with the control group showing unimpeded bacterial growth. Data are presented as mean ± standard deviation (*n* = 3); * *p* < 0.05, ** *p* < 0.01, *** *p* < 0.001.

The antibacterial efficacy of LPTM-3 was systematically evaluated against two clinically relevant pathogens, *Escherichia coli* (*E. coli*, Gram-negative) and *Staphylococcus aureus* (*S. aureus*, Gram-positive), using the paper disk diffusion method and direct contact inhibition assay (Figure 4). Representative images of the inhibition zones (Figure 4A,D) and quantitative analysis (Figure 4B,E) revealed distinct antibacterial potency across all tested groups against both pathogens. The blank paper disk served as a negative control, exhibiting no antibacterial activity. The inhibition zone diameters were measured as the net zone diameter excluding the sample disk. For *E. coli*, PVA-TA showed moderate antibacterial activity with an inhibition zone of 3.59 ± 0.32 mm, while free TA exhibited stronger efficacy at 6.83 ± 0.56 mm, attributed to TA’s inherent polyphenolic antibacterial properties. Notably, LPTM-3 displayed significantly enhanced antibacterial activity against *E. coli*, with an inhibition zone diameter of 4.67 ± 1.13 mm, surpassing PVA-TA and approaching the efficacy of free TA. Against *S. aureus*, a similar trend was observed: PVA-TA achieved an inhibition zone of 4.92 ± 0.75 mm, free TA reached 7.76 ± 0.57 mm, and LPTM-3 exhibited the most balanced and robust performance with an inhibition zone of 5.53 ± 0.67 mm. These results confirm that LPTM-3 retains the antibacterial activity of TA while overcoming the limitations of PVA-TA’s weak inherent antibacterial properties, with the hydrogel network enabling sustained release of TA to exert prolonged antibacterial effects [34].

The direct contact inhibition assay (Figure 4C,F) further validated the practical antibacterial performance of LPTM-3 for wound dressings, which require direct contact with infected tissue. For both E. coli and S. aureus, the control groups (without hydrogel treatment) showed dense bacterial growth across the entire agar surface, indicating unimpeded bacterial proliferation. In stark contrast, the agar plates treated with LPTM-3 exhibited almost no visible bacterial colonies, demonstrating excellent direct contact antibacterial efficacy against both Gram-negative and Gram-positive pathogens. This superior direct contact antibacterial performance arises from the synergistic combination of the hydrogel’s optimized structural and functional properties. The sustained release of TA from LPTM-3’s well-tuned network structure ensures a continuous and controlled delivery of the antibacterial agent to the interface between the hydrogel and bacterial cells [35]. Simultaneously, the robust adhesive strength of LPTM-3 to biological surfaces guarantees intimate and persistent contact with the target area, which maximizes the therapeutic efficacy of the released TA by maintaining proximity to the bacterial population. The complementary results from the paper disk diffusion and direct contact inhibition assays collectively demonstrate that LPTM-3 exhibits antibacterial activity against both Gram-negative (*E. coli*) and Gram-positive (*S. aureus*) pathogens. This dual-pathogen efficacy is a critical functional attribute for wound dressings, where preventing bacterial infection is essential for creating a favorable microenvironment and facilitating subsequent tissue regeneration.

### 2.4. Cytocompatibility and Biocompatibility

**Figure 5:**
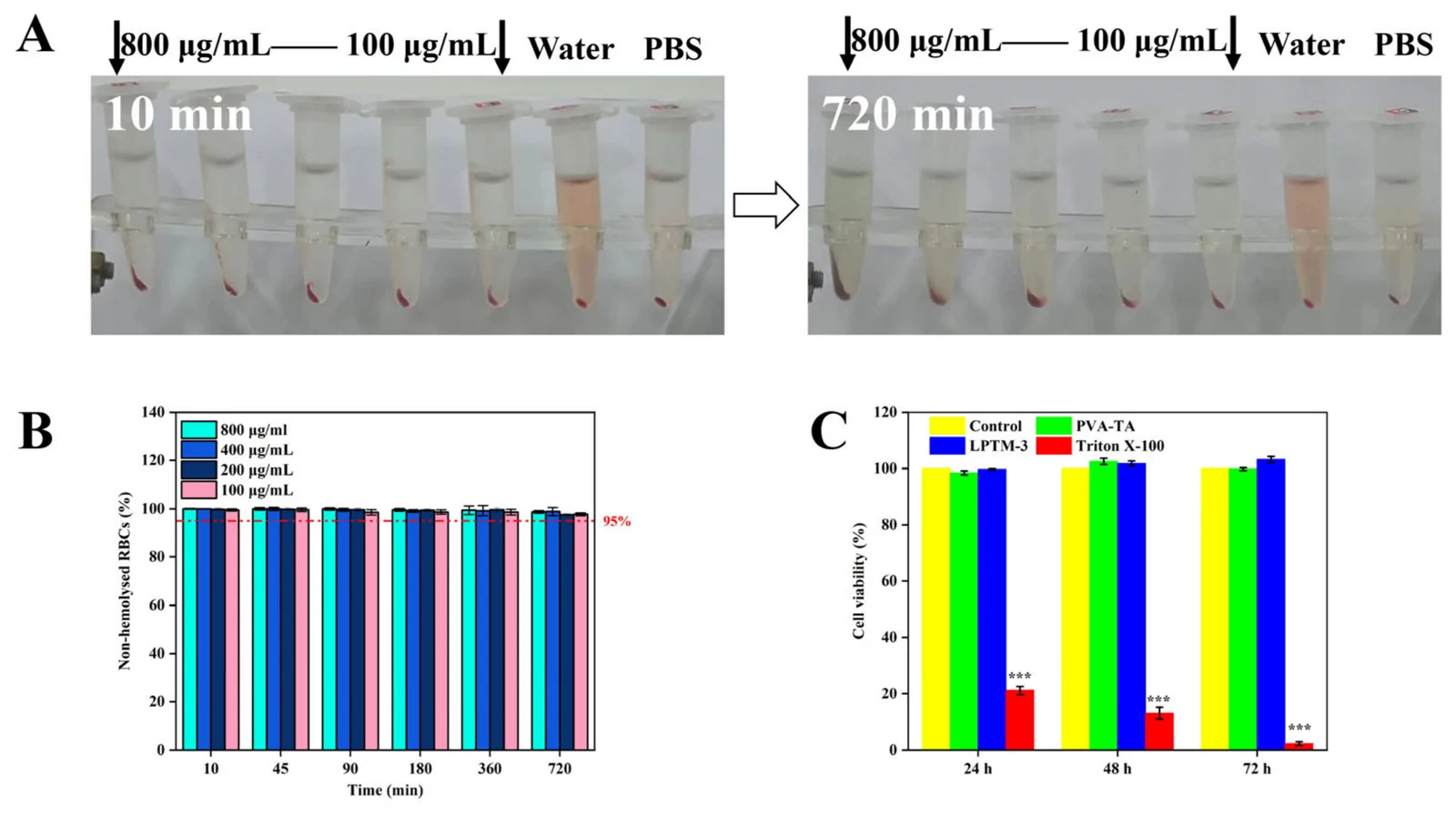
Biocompatibility evaluation of LPTM−3. (**A**) Photographs of hemolysis assay tubes at 10 min and 720 min, showing no visible RBC hemolysis in LPTM−3 groups. (**B**) Quantitative analysis of hemolysis rates at different LPTM−3 concentrations and time points. (**C**) Cell viability assay after incubation with control, PVA−TA, LPTM−3, and Triton X-100 for 24, 48, and 72 h. Data are presented as mean ± standard deviation (*n* = 3); *** *p* < 0.001.

The cytocompatibility and biocompatibility of LPTM-3 were systematically evaluated to assess its safety for in vivo biomedical applications (Figure 5). The hemolysis assay was performed by incubating red blood cells (RBCs) with LPTM-3 extracts at concentrations ranging from 100 to 800 μg/mL over 720 min (Figure 5A,B). At all tested concentrations and time points, the hemolysis rate of LPTM-3 remained well below the internationally accepted threshold of 5% for non-hemolytic materials. Specifically, at the highest concentration of 800 μg/mL, the hemolysis rate was only 0.46 ± 0.45% at 10 min and remained at 2.24 ± 0.59% after 720 min. Even at the lowest concentration of 100 μg/mL, the hemolysis rate was 0.21 ± 0.02% at 10 min and only 1.28 ± 0.57% at 720 min. All values were far below the positive control (water, 100% hemolysis) and approached the negative control (PBS). These results confirm that LPTM-3 exhibits excellent hemocompatibility, with no significant adverse effects on RBC integrity, making it suitable for applications involving blood contact.

The cytocompatibility of LPTM-3 was evaluated using a CCK-8 assay to assess cell viability after 24, 48, and 72 h of incubation (Figure 5C). The control group (untreated cells) and PVA-TA hydrogel showed high cell viability (>90%) at all time points, indicating good baseline biocompatibility. In contrast, the positive control (Triton X-100) induced severe cytotoxicity, with cell viability dropping to <10% at all time points. Notably, LPTM-3 maintained cell viability above 90% at 24 h, 48 h, and 72 h, demonstrating no significant cytotoxicity and even slightly improved viability compared to PVA-TA. This enhanced cytocompatibility is attributed to the optimized network structure of LPTM-3, which enables controlled release of TA without inducing local cytotoxicity, while the LNT component provides additional biocompatible and bioactive properties that support cell proliferation and adhesion [36].

### 2.5. In Vitro Wound Healing and Immunomodulatory Activity

**Figure 6:**
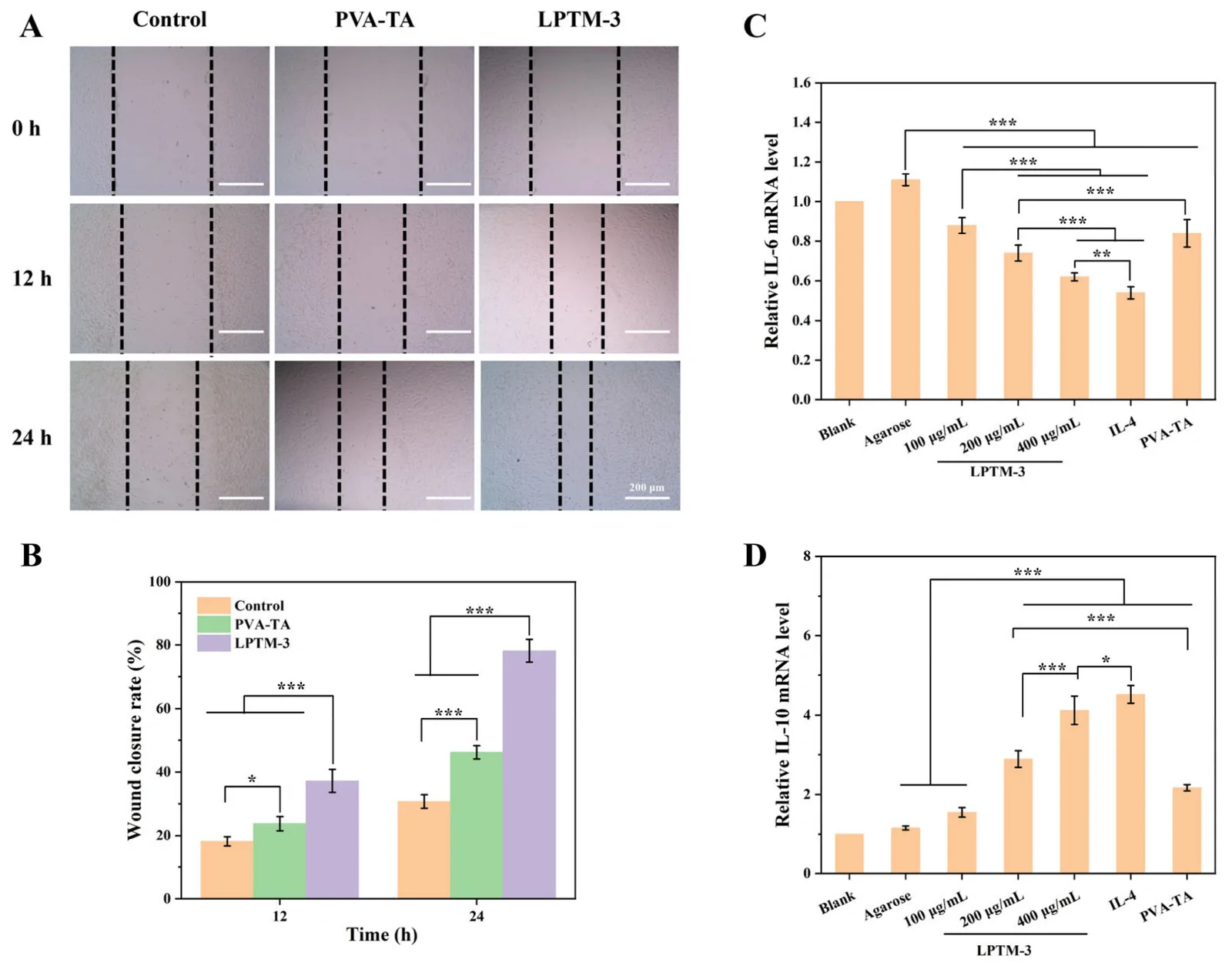
In vitro wound healing and immunomodulatory activity of LPTM−3. (**A**) Representative bright-field images of scratch wound closure in NIH 3T3 fibroblasts treated with Control, PVA−TA, and LPTM−3 extracts at 0, 12, and 24 h (scale bar = 200 μm). (**B**) Quantitative wound closure rates at 12 and 24 h. (**C**,**D**) Relative mRNA expression levels of IL-6 (**C**) and IL-10 (**D**) in RAW 264.7 treated with LNT at different concentrations (100, 200, and 400 μg/mL), PVA−TA (200 μg/mL), and IL-4 (positive control), normalized to the Blank group. Data are presented as mean ± standard deviation (*n* = 3). * *p* < 0.05, ** *p* < 0.01, *** *p* < 0.001.

The pro-migratory effect of LPTM-3 on fibroblasts was evaluated using an in vitro scratch wound healing assay with NIH 3T3 cells (Figure 6A,B). At 12 h, the wound closure rates were 18.14 ± 1.51%, 23.77 ± 2.23%, and 37.23 ± 3.57% for the Control, PVA-TA, and LPTM-3 groups, respectively. By 24 h, LPTM-3 achieved a wound closure rate of 78.13 ± 3.62%, significantly exceeding both the Control (30.75 ± 2.17%) and PVA-TA (46.21 ± 2.09%) groups (*p* < 0.001). PVA-TA also showed a modest improvement over the Control (*p* < 0.05), suggesting that TA release alone provides mild pro-migratory cues, whereas the incorporation of LNT in LPTM-3 further amplifies this effect. These results demonstrate that bioactive LNT released from the hydrogel network actively promotes fibroblast migration, a key cellular process during the proliferative phase of wound repair [37].

To further elucidate the immunomodulatory mechanism underlying the enhanced healing, the effects of LNT on macrophage cytokine expression were assessed by qRT-PCR (Figure 6C,D). LNT treatment down-regulated the pro-inflammatory factor IL-6 in a dose-dependent manner, with the 400 μg/mL group reaching the lowest level (0.62 ± 0.02), while concurrently up-regulating the anti-inflammatory factor IL-10 (4.12 ± 0.36 at 400 μg/mL). Notably, PVA-TA (200 μg/mL) moderately increased IL-10 expression (2.17 ± 0.08) but showed a weaker suppressive effect on IL-6 (0.84 ± 0.04) compared to LNT at the same concentration (IL-6: 0.74 ± 0.04; IL-10: 2.89 ± 0.21). These findings indicate that LNT promotes macrophage polarization toward the reparative M2 phenotype by simultaneously suppressing pro-inflammatory IL-6 and enhancing anti-inflammatory IL-10 secretion, providing a mechanistic basis for the accelerated wound healing observed in vivo [38].

### 2.6. In Vivo Wound Healing Efficacy

**Figure 7:**
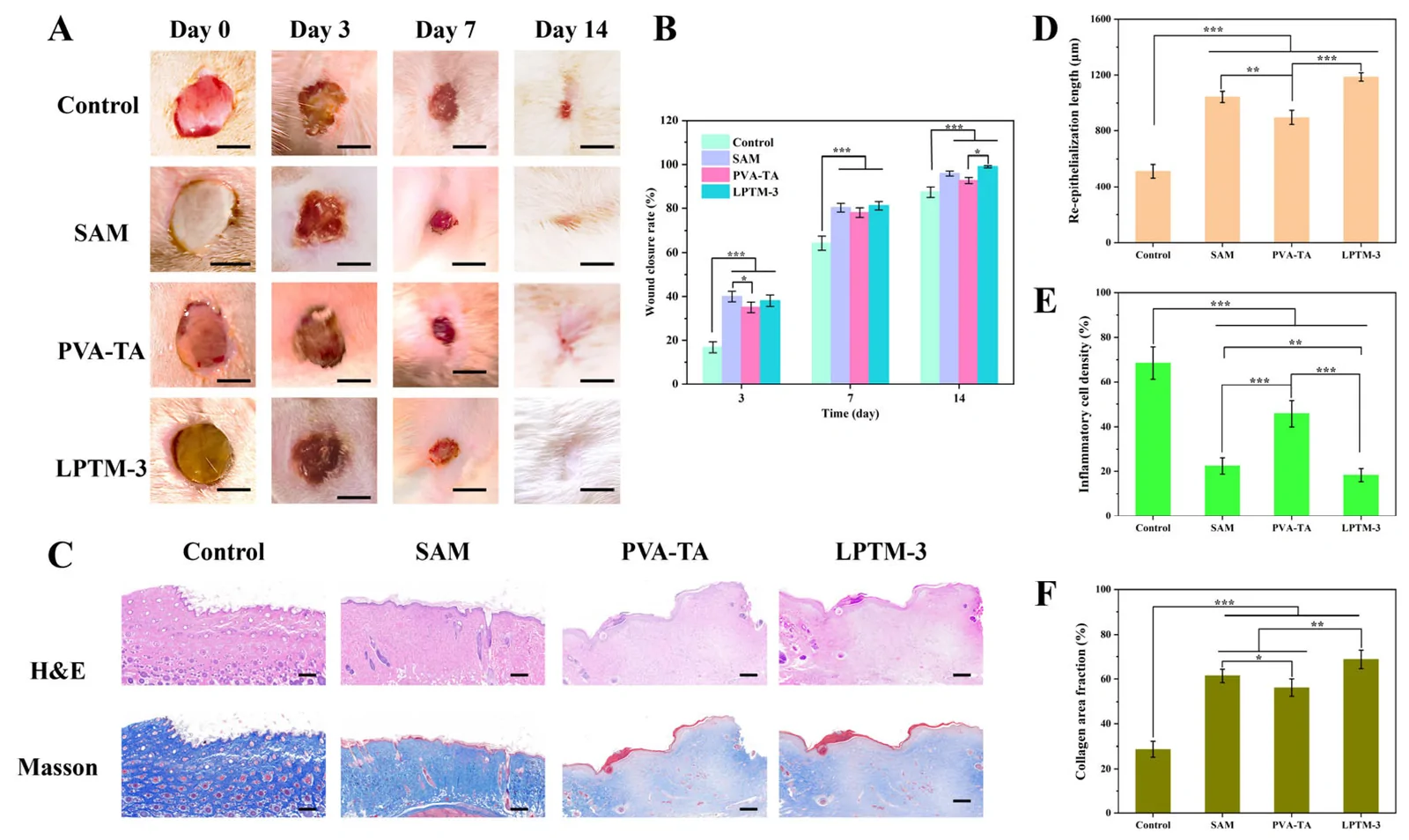
In vivo wound healing efficacy of LPTM−3. (**A**) Representative macroscopic photographs of wound closure over 14 days in Control, SAM, PVA−TA, and LPTM−3 groups (scale bar = 10 mm). (**B**) Quantitative analysis of wound closure rates over 14 days. (**C**) H&E and Masson staining of wound tissue sections at day 14 (scale bar = 200 μm). (**D**) Re-epithelialization length quantification and (**E**) Inflammatory cell density analysis (cells per high-power field, HPF) from H&E-stained sections. (**F**) Collagen deposition area fraction from Masson-stained sections. Data are presented as mean ± standard deviation (*n* = 4). * *p* < 0.05, ** *p* < 0.01, *** *p* < 0.001.

The in vivo wound healing performance of LPTM-3 was evaluated using a full-thickness skin defect model in rats, with an untreated Control, a commercial Silver Antimicrobial Dressing (SAM), a widely used clinical wound care product, and PVA-TA hydrogel as reference groups (Figure 7).

Representative photographs of wound healing over 14 days (Figure 7A) and quantitative analysis of wound closure rates (Figure 7B) revealed significant differences in healing efficacy across groups. The Control group showed the slowest wound closure, with only 16.81 ± 2.50% closure by day 3 and 87.31 ± 2.13% closure by day 14, indicating limited spontaneous healing. In contrast, the commercial SAM and PVA-TA groups exhibited moderate healing, reaching 40.01 ± 2.41% and 35.05 ± 2.27% closure by day 3, respectively, and 95.93 ± 1.12% and 92.73 ± 1.40% closure by day 14. Notably, the LPTM-3 group demonstrated the most rapid and complete wound closure: 38.08 ± 2.60% closure by day 3, 81.08 ± 1.90% closure by day 7, and nearly complete closure (99.06 ± 0.32%) by day 14, with the most notable advantage observed during the day 3–7 acceleration phase. This accelerated healing is attributed to the synergistic effects of LPTM-3’s multifunctional properties: sustained moisture retention, controlled TA release for antioxidant and antibacterial protection, and a flexible, biocompatible network that supports tissue regeneration [39]. Specifically, the sustained release of TA provides continuous antioxidant protection that mitigates excessive reactive oxygen species at the wound site, thereby facilitating the timely transition from the inflammatory phase to the proliferative phase. Meanwhile, LNT-derived bioactive components may modulate local immune responses by promoting macrophage polarization toward the reparative M2 phenotype, collectively contributing to the accelerated healing observed in the LPTM-3 group.

Histological analysis (Figure 7C) further confirmed the superior healing efficacy of LPTM-3. H&E staining revealed that the Control group exhibited incomplete re-epithelialization and persistent inflammation, while the SAM and PVA-TA groups showed moderate re-epithelialization with reduced inflammatory cell infiltration. In contrast, the LPTM-3 group displayed nearly complete re-epithelialization, well-organized granulation tissue, and minimal inflammatory cell infiltration, indicating advanced tissue regeneration. Masson staining confirmed that LPTM-3 promoted substantial collagen deposition with more mature and organized collagen fiber architecture compared to other groups.

To objectively quantify these histological observations, image analysis was performed on H&E and Masson’s trichrome-stained sections. Quantitative analysis of re-epithelialization length (Figure 7D) showed that the LPTM-3 group achieved the longest epithelial coverage (1186.75 ± 29.53 μm), significantly exceeding the Control (512.33 ± 48.67 μm), SAM (1043.25 ± 39.84 μm), and PVA-TA (896.50 ± 52.31 μm) groups. Inflammatory cell density analysis (Figure 7E) revealed that the LPTM-3 group had the lowest inflammatory cell infiltration (18.25 ± 2.94 cells/HPF), significantly lower than the Control (68.25 ± 7.23 cells/HPF), PVA-TA (45.75 ± 5.81 cells/HPF), and SAM (22.33 ± 3.67 cells/HPF) groups. Collagen deposition area fraction quantification (Figure 7F) demonstrated that the LPTM-3 group exhibited the highest collagen content (68.73 ± 4.12%), significantly greater than the Control (28.67 ± 3.45%) and comparable to the SAM group (61.42 ± 2.89%), with more organized fiber alignment as observed in Masson staining. These quantitative histological results corroborate the macroscopic wound closure data, demonstrating that LPTM-3 not only accelerates wound closure but also improves the quality of tissue repair.

Collectively, the in vivo wound healing results demonstrate that LPTM-3 significantly accelerates full-thickness skin defect repair, promoting rapid re-epithelialization, collagen deposition, and tissue regeneration. This accelerated healing is attributed to the synergistic combination of sustained TA release avoiding burst cytotoxicity, LNT-derived immunomodulatory bioactivity, superior moisture retention, and balanced mechanical conformability. This superior healing efficacy, combined with its excellent biocompatibility, antibacterial, and antioxidant properties, validates LPTM-3 as a highly promising candidate for next-generation wound dressing applications, with potential to match or slightly exceed existing commercial products like SAM. It should be noted that the present study evaluated wound healing efficacy in a non-infected full-thickness defect model; future investigations employing infected wound models are warranted to further elucidate the contribution of the antibacterial components to wound repair.

## 3. Conclusions

This study successfully developed a multifunctional lentinan–polyvinyl alcohol–tannic acid (LPTM) composite hydrogel for wound dressing applications, with LPTM-3 identified as the optimal formulation. Distinctive from conventional hydrogels, LPTM-3 forms a stable, homogeneous cross-linked network via hydrogen bonding, exhibiting balanced physicochemical properties including appropriate swelling, superior water retention, and biodegradability suitable for temporary biomedical use. It achieves an optimal balance of mechanical strength and ductility, enabling flexibility for wearable compatibility while maintaining structural integrity. A key feature is its sustained TA release kinetics, avoiding cytotoxicity and ensuring prolonged bioavailability, which confers robust antioxidant activity and antibacterial efficacy against both Gram-negative and Gram-positive pathogens. LPTM-3 also demonstrates excellent biocompatibility with low hemolysis and high cell viability, confirming its safety for in vivo applications. Most notably, in a rat full-thickness skin defect model, LPTM-3 accelerates wound closure and achieves comparable or slightly superior wound closure to a commercial silver antimicrobial dressing, with notably accelerated closure during the intermediate phase, accompanied by enhanced re-epithelialization and collagen deposition. Collectively, LPTM-3 integrates controlled bioactive release, mechanical adaptability, biocompatibility, and superior in vivo healing efficacy, serving as a promising candidate for next-generation wound dressings and providing a rational design strategy for multifunctional polysaccharide-based biomaterials.

## 4. Materials and Methods

### 4.1. Materials

Lentinan (LNT) derived from Lentinus edodes fruit bodies with a molecular weight of 300 kDa and purity of 99.0% was sourced from Tianbao Biotechnology Ltd. (Xi’an, China). Polyvinyl alcohol (PVA), tannic acid (TA), 1,1-diphenyl-2-picrylhydrazyl (DPPH), glycerol and other analytical grade chemical reagents were purchased from Shanghai Macklin Biochemical Co., Ltd. (Shanghai, China). All biological culture reagents including Luria Bertani (LB) medium, Dulbecco’s Modified Eagle Medium (DMEM), fetal bovine serum (FBS), Cell Counting Kit 8 (CCK-8) and lysozyme were obtained from Beijing Solarbio Science & Technology Co., Ltd. (Beijing, China). *Escherichia coli* strain (*E. coli*, ATCC 25922) and *Staphylococcus aureus* strain (*S. aureus*, ATCC 25923) were provided by Guangdong Microbial Culture Collection Center (Guangzhou, China). NIH 3T3 mouse embryonic fibroblast cells (ATCC CRL-1658; RRID: CVCL_0594) and RAW 264.7 mouse monocyte-macrophage cells (ATCC TIB-71; RRID: CVCL_0493) were kindly provided by Dr. Botao Gao from the Institute of Biological and Medical Engineering, Guangdong Academy of Sciences (Guangzhou, China). Female Sprague Dawley (SD) rats were provided by Shulaibao Co., Ltd. (Wuhan, China). All reagents were used without further purification unless otherwise specified.

### 4.2. Preparation of LPTM Hydrogels

The composite hydrogels were synthesized via a freeze–thaw-assisted immersion method. Briefly, LNT and PVA were respectively dissolved in deionized water to form 3.5 wt% and 10 wt% aqueous solutions, with LNT dissolved at 70 °C and PVA dissolved at 95 °C under continuous magnetic stirring. The two solutions were then mixed at predetermined mass ratios (LNT:PVA = 1:3, 1:1, and 3:1 for LPTM-1, LPTM-2, and LPTM-3, respectively), and supplemented with approximately 0.1 mL of glycerol (~30 wt% relative to total polymer mass) as a plasticizer. The mixture was homogenized at 10,000 rpm for 5 min using a high-speed shear homogenizer, then centrifuged at 3000 rpm for 10 min at room temperature to remove air bubbles, cast into Petri dishes, and air-dried at room temperature to form films. Subsequently, the films were immersed in deionized water for 1 min to fully wet the surface, facilitating the formation of a uniform physically cross-linked network during freeze–thaw cycles, and then subjected to 5 freeze–thaw cycles (24 h at −20 °C, followed by 4 h thawing) to induce physical cross-linking. The resulting LNT-PVA hydrogels were immersed in a 10% (*w*/*v*) TA aqueous solution at 25 °C for 48 h to incorporate TA via hydrogen bonding, rinsed three times with deionized water (~10 s each) to remove surface-bound unreacted TA, dried at room temperature to constant weight (residual moisture content ~10%), and sealed in bags for storage. The TA loading content in the final LPTM-3 films was determined to be approximately 62 mg/g.

### 4.3. Physicochemical Characterization of Hydrogels

#### 4.3.1. Swelling Ratio Measurement

Hydrogel swelling behavior was characterized gravimetrically. Dry hydrogel samples (initial weight W_0_) were submerged in PBS (pH 7.4, 20 mL) maintained at 37 °C. At specified intervals (30–180 min), specimens were retrieved, gently blotted to remove excess surface moisture, and weighed (W_t_) promptly. Swelling capacity was expressed as: SR (%) = (W_t_ − W_0_)/W_0_ × 100%, with each formulation tested in three independent replicates.

#### 4.3.2. Water Retention Capacity Assay

To assess water retention ability, hydrogels were first allowed to swell to equilibrium in PBS (37 °C, pH 7.4). After removing surface liquid by blotting, the fully swollen mass (W_s_) was documented. Samples were then kept under ambient conditions, and their weights (W_r_) were monitored over 180 min at 30 min intervals. Water retention was quantified as: WRC (%) = W_r_/W_s_ × 100%, with three parallel measurements per group.

#### 4.3.3. In Vitro Degradation Rate Analysis

Degradation kinetics were examined in lysozyme-supplemented PBS (0.01 mg/mL, pH 7.4) under mild agitation at 37 °C. Pre-weighed dry hydrogels (Wd_0_) were placed in the enzymatic solution, and at selected time points (60–300 min), specimens were withdrawn, washed with deionized water, freeze-dried to constant mass (Wd_t_), and weighed. Mass loss was determined by: Degradation rate (%) = (Wd_0_ − Wd_t_)/Wd_0_ × 100%, with triplicate measurements for each time point.

#### 4.3.4. Fourier Transform Infrared (FTIR) Spectroscopy

FTIR was used to characterize the chemical structure and intermolecular interactions of the hydrogels. Measurements were performed on a FTIR spectrometer (Thermo Fisher Scientific, Waltham, MA, USA) in the attenuated total reflection (ATR) mode. The spectra of pure LNT, PVA, TA, and the optimized LPTM-3 hydrogel were collected over the wavenumber range of 4000–500 cm^−1^ with a resolution of 4 cm^−1^ and 32 scans per sample.

#### 4.3.5. Mechanical Tensile Testing

The mechanical properties of the hydrogels were evaluated using a universal testing machine (UTM, Instron 5967, Norwood, MA, USA) at room temperature. Hydrogel samples were cast into a custom mold to control the thickness at 1 mm, then cut into rectangular strips (20 mm × 10 mm × 1 mm) with an initial gauge length of 10 mm between the fixtures, and subjected to uniaxial tension at a crosshead speed of 5 mm/min until fracture. The samples were equilibrated in deionized water at room temperature for 2 h prior to testing. Tensile strength, elongation at break, and tensile modulus were calculated from the stress–strain curves. All tests were performed in triplicate for each formulation.

#### 4.3.6. Adhesive Strength Measurement

Adhesive strength on porcine skin was determined using a lap-shear test. Fresh porcine skin was obtained from the dorsal region, deprived of hair and subcutaneous fat, cleaned with PBS, and cut into 30 mm × 30 mm pieces. The skin was mounted on the testing platform, and hydrogel samples (10 mm × 10 mm) were wetted with deionized water for 1 min prior to application on the skin surface with a constant pressure of 5 N for 1 min. The samples were then pulled at a rate of 1 mm/min, and the maximum force required to detach the hydrogel was recorded. Adhesive strength was calculated as the maximum force divided by the contact area. For qualitative assessment of substrate adhesion versatility, LPTM-3 hydrogel samples (10 mm × 10 mm) were manually applied to six distinct substrates (bone, muscle, carton, plastic, glass, and porcine skin) under uniform manual pressure, and the adhesion behavior was visually recorded by a digital camera. All quantitative tests were conducted in triplicate.

### 4.4. In Vitro TA Release Kinetics

Pre-weighed dry hydrogel samples (20 mg) were immersed in 20 mL of PBS (pH 7.4) at 37 °C under gentle shaking at 100 rpm. At predetermined time points (15, 30, 60, 90, 120, and 150 min), 1 mL of the release medium was withdrawn and an equal volume of fresh pre-warmed PBS (pH 7.4, 37 °C) was immediately added to maintain sink conditions. The concentration of released TA was determined by the Folin–Ciocalteu colorimetric method at 760 nm as described previously [40]. A calibration curve of TA (0–50 μg/mL, n = 3) yielded the regression equation A = 0.0206C + 0.0005 (r^2^ = 0.9985). The LOD and LOQ, determined as 3.3S_y/x/s and 10S_y/x/s, were 2.66 and 8.05 μg/mL, respectively, confirming adequate sensitivity for quantifying TA release. Specificity was verified using PBS blanks and TA-free PVA-LNT extracts, which showed no detectable interference at 760 nm, confirming that the measured signal was attributable to released TA. All experiments were performed in triplicate.

### 4.5. DPPH Radical Scavenging Assay

Free radical scavenging capacity was evaluated via the DPPH assay. Briefly, hydrogel samples (10 mg) were extracted in methanol (10 mL) for 24 h with gentle agitation. The resulting extract (2 mL) was combined with an equal volume of DPPH methanol solution (0.1 mM), and the reaction mixture was kept in darkness for 30 min. Absorbance readings at 517 nm were recorded, and radical scavenging efficiency was computed as: Scavenging rate (%) = (A_0_ − A_1_)/A_0_ × 100%, where A_0_ and A_1_ represent the absorbance of the blank (DPPH solution alone) and sample, respectively. Salicylic acid served as the reference antioxidant, with all assays performed in triplicate.

### 4.6. Antibacterial Activity Assays

The antibacterial activity of the hydrogels was evaluated against two clinically relevant pathogens: *E. coli* (Gram-negative) and *S. aureus* (Gram-positive), using two complementary methods: the inhibition zone assay and the direct contact inhibition assay.

#### 4.6.1. Inhibition Zone Assay

Bacterial strains were cultured in LB broth at 37 °C under orbital shaking (180 rpm) to mid-log phase (OD_600_ ≈ 0.1, ~10^7^ CFU/mL). A 100-μL aliquot of each bacterial suspension was uniformly distributed onto LB agar plates. Hydrogel discs (6 mm diameter), TA-loaded filter papers (10% *w*/*v*), and blank discs (negative control) were UV-sterilized (30 min) and placed on the inoculated surfaces. Following 24 h incubation at 37 °C, inhibition zone diameters were measured with a digital caliper, excluding the disc area. Each assay was conducted with three replicates.

#### 4.6.2. Viable Bacteria Colony Counting Assay

The quantitative antibacterial efficacy of the hydrogels was determined via the colony-forming unit (CFU) counting method. Briefly, the activated bacterial suspension was diluted to a final concentration of 1 × 10^6^ CFU/mL with sterile LB medium. Sterilized LPTM-3 hydrogel samples (10 mg) were added into 1 mL of the prepared bacterial suspension, and the mixture was incubated at 37 °C with shaking at 180 rpm for 24 h. The bacterial suspension without hydrogel treatment was set as the blank control group. After incubation, the bacterial suspension was serially diluted 10-fold with sterile PBS to a gradient of 10^−1^ to 10^−6^, and 100 μL of the 10^−5^ dilution was spread evenly on solid LB agar plates. All plates were incubated upside down at 37 °C for 24 h, and the number of visible colonies on each plate was counted. All experiments were conducted in triplicate.

### 4.7. In Vitro Biocompatibility Assays

The in vitro biocompatibility of the hydrogels was systematically evaluated via a hemolysis assay and a cell cytotoxicity assay, to assess the blood compatibility and cytocompatibility for wound dressing applications.

#### 4.7.1. Hemolysis Assay

Hemocompatibility was assessed using rat erythrocytes. Fresh blood collected from SD rats was anticoagulated with sodium citrate, and RBCs were isolated by centrifugation (3000 rpm, 10 min), washed thrice with PBS, and resuspended to 5% (*v*/*v*). Hydrogel extracts (sterilized samples incubated in PBS at 37 °C for 24 h) were diluted to 100–800 μg/mL. Each extract (0.5 mL) was combined with an equal volume of RBC suspension, with PBS and water serving as negative and positive controls, respectively. Following incubation at 37 °C for various durations (10–720 min), mixtures were centrifuged (3000 rpm, 10 min), and supernatant absorbance was read at 540 nm. Hemolysis percentage was calculated from these measurements, with all experiments performed in triplicate.

#### 4.7.2. In Vitro Cytotoxicity Assay

The cytotoxicity of the hydrogels was evaluated using the CCK-8 assay with NIH 3T3 fibroblast cells. NIH 3T3 cells were cultured in DMEM supplemented with 10% FBS and 1% penicillin-streptomycin, in a humidified incubator at 37 °C with 5% CO_2_. Sterilized hydrogel samples were immersed in complete DMEM medium at a ratio of 0.1 g/mL, and incubated at 37 °C for 24 h to prepare hydrogel extracts, which were then filtered through a 0.22 μm sterile filter for subsequent use. NIH 3T3 cells were seeded into 96-well plates at a density of 1 × 10^4^ cells per well and incubated for 24 h to allow cell attachment. After attachment, the original culture medium was removed, and 100 μL of fresh complete medium (blank control group), PVA-TA hydrogel extract, LPTM-3 hydrogel extract, or 0.1% Triton X-100 (positive control group) was added to each well, with 3 replicate wells set for each group. The plates were incubated for 24, 48, and 72 h, respectively. At each predetermined time point, the medium in each well was replaced with 100 μL of serum-free DMEM containing 10% CCK-8 reagent, and the plates were incubated for another 2 h at 37 °C. The absorbance of each well was measured at 450 nm using a microplate reader. The relative cell viability was calculated using the formula: Cell viability (%) = (OD_sample − OD_blank)/(OD_control − OD_blank) × 100%, where OD_sample is the absorbance of the experimental group, OD_control is the absorbance of the blank control group, and OD_blank is the absorbance of the medium without cells. All experiments were repeated three times independently.

### 4.8. Scratch Wound Healing Assay

NIH 3T3 cells were seeded into 6-well plates at a density of 5 × 10^5^ cells per well and cultured in complete DMEM at 37 °C with 5% CO_2_ until reaching ~95% confluence. A uniform scratch was created across the center of each well using a sterile 200 μL pipette tip. After washing twice with PBS to remove detached cells, hydrogel extracts were prepared by immersing sterilized LPTM-3 or PVA-TA samples (0.1 g) in 10 mL of serum-free DMEM at 37 °C for 24 h, followed by 0.22 μm filtration. The scratched monolayers were treated with serum-free DMEM (Control), PVA-TA extract, or LPTM-3 extract. Bright-field images were captured at 0, 12, and 24 h using an inverted microscope (BX53, Olympus, Tokyo, Japan). The wound closure rate was calculated as: Wound closure rate (%) = (W_0_ − W_t_)/W_0_ × 100%, where W_0_ and W_t_ represent the scratch width at 0 h and the corresponding time point, respectively.

### 4.9. Quantitative Real-Time PCR (qRT-PCR)

RAW 264.7 mouse monocyte-macrophage cells were cultured in DMEM supplemented with 10% FBS and 1% penicillin-streptomycin at 37 °C with 5% CO_2_. Cells were seeded into 6-well plates at a density of 5 × 10^5^ cells per well and allowed to attach overnight. Subsequently, the cells were treated with LNT (100, 200, and 400 μg/mL), PVA-TA (200 μg/mL), or IL-4 (20 ng/mL, positive control) for 24 h. Agarose, as an inert polysaccharide, was used as a negative polysaccharide control. Total RNA was extracted using TRIzol reagent (Invitrogen, Carlsbad, CA, USA) and reverse-transcribed into cDNA using a reverse transcription kit (TaKaRa, Kyoto, Japan) according to the manufacturer’s protocols. qRT-PCR was performed on a real-time PCR system (Applied Biosystems, Carlsbad, CA, USA) using SYBR Green Master Mix. The relative mRNA expression levels of IL-6 and IL-10 were calculated by the 2^(−ΔΔCt) method, with β-actin as the internal reference gene. Primer sequences were as follows:

IL-6 forward 5′-CAATAGAAGACTGTGAGCATC-3′ and reverse 5′-CCCATAATCAGCCACCAAACC-3′;

IL-10 forward 5′-CTTAGAGCCACCCAACAAATAC-3′ and reverse 5′-AGAGACAGATGAGCAAGAGAC-3′;

β-actin forward 5′-ACCCAGATCATGTTTGAGACC-3′ and reverse 5′-GATCTTCATGAGGTAGTCTGTC-3′.

### 4.10. In Vivo Full-Thickness Skin Wound Healing Assay

All animal experimental procedures were approved by the Ethics Committee of Hubei Engineering University and performed in strict accordance with the Guide for the Care and Use of Laboratory Animals.

#### 4.10.1. Animal Model Establishment and Grouping

Female SD rats, weighing 200 ± 20 g, were used in this study. All rats were housed in a constant temperature (25 °C) and 50% humidity environment with a 12 h light/dark cycle, with free access to sterile food and water, and acclimatized for 1 week before the experiment. Rats were randomly divided into 4 groups (n = 4 per group) using a random number table. This sample size was selected based on commonly accepted standards for preliminary animal wound healing studies, balancing statistical validity with the 3R principle of animal experimentation: (1) Blank control group (Control): the wound was only covered with sterile gauze; (2) Commercial dressing group: the wound was covered with a commercial Silver Antimicrobial Dressing (SAM); (3) PVA-TA hydrogel group: the wound was covered with PVA-TA hydrogel; (4) LPTM-3 hydrogel group: the wound was covered with the optimized LPTM-3 composite hydrogel.

For wound model establishment, rats were anesthetized via intraperitoneal injection of 5% sodium pentobarbital at a dose of 30 mg/kg body weight. The dorsal hair was removed, and the skin was disinfected with iodophor. Under sterile conditions, a full-thickness skin defect wound with a diameter of 10 mm was created on both sides of the dorsal spine of each rat using a sterile biopsy punch, with the depth reaching the fascial layer. After complete hemostasis, the corresponding treatments were applied to each group, and all wounds were fixed with sterile breathable adhesive dressings (3M Tegaderm, Brookings, SD, USA), which were replaced every 2 days. Prior to each dressing replacement, the wound bed was gently cleansed with sterile saline to remove residual debris. No additional wound bed debridement was performed throughout the study. The hydrogel patches were applied directly onto the wound bed, conforming to the wound surface without suturing.

#### 4.10.2. Wound Closure Evaluation

The wounds were photographed with a digital camera at standardized positions on Day 0, 3, 7, and 14 after surgery. The wound area at each time point was measured using ImageJ1.54d software (NIH, Bethesda, MD, USA). Photographs were acquired at a fixed distance of 15 cm with a standardized scale bar for calibration. The wound edge was manually traced using the freehand selection tool, and the enclosed area was automatically calculated. Measurements were performed by a single investigator to minimize inter-observer variability. The wound closure rate was calculated using the following formula:

Wound closure rate (%) = (A_0_ − A_t_)/A_0_ × 100%

where A_0_ was the initial wound area on Day 0, and A_t_ was the residual wound area at the corresponding time point.

#### 4.10.3. Histological Analysis

On Day 14 after surgery, all rats were euthanized via intraperitoneal injection of an overdose of 5% sodium pentobarbital (150 mg/kg body weight). The full-thickness wound tissue and surrounding normal skin were harvested immediately after euthanasia, fixed in 4% paraformaldehyde for 24 h, dehydrated with a graded ethanol series (70%, 80%, 90%, 95%, and 100% ethanol, 15 min per gradient), cleared with xylene, and embedded in paraffin wax. Serial paraffin sections with a thickness of 4 μm were prepared for hematoxylin-eosin (H&E) staining and Masson’s trichrome staining following standard histopathological protocols. The re-epithelialization, inflammatory cell infiltration, collagen deposition, and tissue remodeling of the wound tissue were observed and photographed under an optical microscope (BX53, Olympus, Tokyo, Japan) at 100× magnification.

### 4.11. Statistical Analysis

All data are expressed as mean ± standard deviation (SD) from at least three independent experiments. Statistical analyses were performed using OriginPro 2024 (OriginLab, Northampton, MA, USA). Normality and homogeneity of variance were assessed using the Shapiro–Wilk and Levene tests, respectively.

For independent multi-group comparisons of physicochemical properties (Figure 1C–E, Figure 2D,E and Figure 3B), TA release (Figure 3C), DPPH scavenging (Figure 3D), antibacterial inhibition zones (Figure 4B,E), hemolysis (Figure 5B), cell viability (Figure 5C), scratch wound closure (Figure 6B), and qRT-PCR cytokine expression (Figure 6C,D), one-way analysis of variance (ANOVA) followed by Tukey’s honestly significant difference (HSD) post hoc test was applied when data met normality and homoscedasticity assumptions; otherwise, the non-parametric Kruskal–Wallis test followed by Dunn’s multiple comparison test was used.

The in vivo wound closure rates measured on days 0, 3, 7, and 14 (Figure 7B) were analyzed by two-way repeated-measures ANOVA, with treatment group and time as factors, followed by Bonferroni post hoc correction for multiple comparisons. The histological semi-quantitative endpoints, including re-epithelialization length, inflammatory cell density, and collagen deposition area fraction (Figure 7D–F), were compared across groups using the Kruskal–Wallis test followed by Dunn’s multiple comparison test.

All statistical tests were two-sided. Differences were considered statistically significant at *p* < 0.05, with significance levels denoted as * *p* < 0.05, ** *p* < 0.01, and *** *p* < 0.001.

## Figures and Tables

**Table 1:** Kinetic parameters for TA release from PVA−TA and LPTM hydrogels.

Samples	Zero-Order		First-Order		Higuchi kH		Korsmeyer–Peppas		
	k_0_	r^2^	k_1_	r^2^	k_H_	r^2^	k_KP_	n	r^2^
PVA-TA	0.6257	0.8907	0.0130	0.8235	12.829	0.8716	0.0002	1.716	0.9136
LPTM-1	0.4153	0.9146	0.0066	0.8305	7.819	0.8267	0.0037	1.072	0.8944
LPTM-2	0.3416	0.9585	0.0047	0.9185	6.612	0.9150	0.0016	1.266	0.9634
LPTM-3	0.3058	0.9674	0.0041	0.9308	5.784	0.9182	0.0026	1.170	0.9784

## Data Availability

The data presented in this study are available on request from the corresponding author.

## Abbreviations

The following abbreviations are used in this manuscript:

LNT	Lentinan
PVA	Polyvinyl alcohol
TA	Tannic acid
LPTM	Lentinan–polyvinyl alcohol–tannic acid membrane
ROS	Reactive oxygen species
PBS	Phosphate-buffered saline
DPPH	1,1-diphenyl-2-picrylhydrazyl
CFU	Colony-forming unit
DMEM	Dulbecco’s Modified Eagle Medium
FBS	Fetal bovine serum
CCK-8	Cell Counting Kit-8
SA	Salicylic acid
*E. coli*	*Escherichia coli*
*S. aureus*	*Staphylococcus aureus*
RBC	Red blood cell
H&E	Hematoxylin-Eosin
SAM	Silver Antimicrobial Dressing
SD	Sprague Dawley
